# News from the melanoma sessions of the European Cancer Congress 2017

**DOI:** 10.1186/s12916-017-0826-4

**Published:** 2017-03-17

**Authors:** Piotr Rutkowski, Katarzyna Kozak

**Affiliations:** Department of Soft Tissue/Bone Sarcoma and Melanoma; Maria Sklodowska-Curie Memorial Cancer Center and Institute of Oncology, Roentgena 5, Warsaw, 02-781 Poland

**Keywords:** Melanoma, Ipilimumab, Adjuvant, Neoadjuvant, BRAF inhibitor, Pembrolizumab, Anti-PD-1

## Abstract

During the European Cancer Congress, the melanoma sessions focused on practice changing trials. Recent developments and approvals in immunotherapy and targeted agents have significantly changed the landscape of melanoma therapy in the metastatic setting and provide great promise for adjuvant and neoadjuvant treatment in high-risk locoregional disease. Perioperative (combined pre- and postoperative) strategies may be extremely beneficial for patients with bulky stage IIIC disease. The long-term results of the European Organisation for Research and Treatment of Cancer (EORTC) 18071 adjuvant trial ipilimumab versus placebo after complete resection of high-risk stage III melanoma, demonstrating improvement in overall survival, has established the reference bar for further trials with postoperative therapy.

## Background

Melanoma accounts for a small percentage of all skin malignancies, but it is still responsible for a majority of deaths due to cutaneous cancers. Moreover, because of aging and an increasing population, the age at death of melanoma patients has steadily increased, with present predictions showing that the number of melanoma cases will continue to increase until 2030, after which time we may see a decrease [1]. Nevertheless, recent developments and approvals in immunotherapy and targeted agents have significantly changed the landscape of melanoma therapy in the metastatic setting and provide great promise for adjuvant and neoadjuvant treatment in high-risk locoregional disease [2–5].

The European Cancer Congress took place on January 27–30, 2017, in Amsterdam, The Netherlands, with a main focus on practice changing trials and oncopolicy. The aim of this article was to describe the highlights from the Melanoma Sessions, which addressed mainly present and future use of adjuvant and neoadjuvant treatment in high-risk locoregional disease as well as studies on immunotherapy in poorly prognostic subgroups of metastatic patients, which are important from a practical point of view.

## Adjuvant therapy

One of the most important issues was the debate on the long-term results of the European Organisation for Research and Treatment of Cancer (EORTC) 18071 adjuvant trial ipilimumab versus placebo after complete resection of high-risk stage III melanoma. It is the first trial with an immune checkpoint inhibitor used postoperatively after lymph node dissection, showing significant improvement in recurrence-free survival and overall survival (5-year RFS rates 40.8% vs. 30.3% and 5-year OS rates 65.4% vs. 54.4%, respectively) [6]. Despite clear benefits in reduction of risk of death, the use of adjuvant ipilimumab remains controversial considering the significant adverse event rates – grade 3–4 immune-related adverse events occurred in 41.6% of patients treated with ipilimumab as compared to 2.7% in placebo arm, resulting in only 42% of patients receiving more than four doses of ipilimumab. Further dilemmas were presented during the debate “This house believes that high-risk stage III melanoma should NOT be treated with adjuvant ipilimumab after complete resection” by Prof. A. Eggermont, Dr A. van Akkooi, Prof. D. Schadendorf, Prof. G. Long, Prof. J. Haanen, Prof. P. Nathan, concerning the ipilimumab dose and duration of treatment as well as patient selection. Over the next 2 years, the landscape of adjuvant therapy will likely be significantly changed following the reporting of results of trials with BRAF (+ MEK) inhibitors and anti-PD-1 drugs.

## Neoadjuvant treatment

Another block of presentations regarded neoadjuvant therapy of locally advanced melanomas. Some preliminary results of trials with BRAF and MEK inhibitors were presented.

Prof. Haanen presented the first results of the Reductor trial on 13 patients with initially unresectable or locally advanced (with high chance of a positive margin) *BRAF*-positive stage IIIC melanoma [7]. Patients were treated with dabrafenib and trametinib for 8 weeks; 11 patients underwent surgery and, finally, 9 achieved R0 resection (9/13 = 69% R0 resectability rate). Of these 9 patients (with median follow-up of 9 months; range 3–22 months), 6 had a recurrence and 3 remain disease free.

Dr Amaria discussed a trial of neoadjuvant (8 weeks) plus adjuvant (44 weeks) therapy with dabrafenib and trametinib versus standard upfront surgery [8]. In 21 patients, neoadjuvant and adjuvant dabrafenib/trametinib (14 patients) significantly improved relapse-free survival over the standard arm (7 patients; *P* < 0.0001). Among 12 patients assessed after preoperative therapy, complete pathological response was observed in 7 (58%).

Prof. Long presented the results of a trial with neoadjuvant and adjuvant dabrafenib plus trametinib combination in *BRAF*-mutated stage III bulky disease resectable melanoma patients who were treated preoperatively for 12 weeks and postoperatively up to 52 weeks [9]. In 30 assessed patients after 12 weeks of therapy, pathological and PET-FDG metabolic complete response was achieved in 50%. Nine patients (30%) recurred during follow-up, with a median time to recurrence of 51 weeks.

These results of neoadjuvant targeted therapy for locally advanced BRAF-mutated stage IIIC melanomas seems promising. On the other hand, the OpACIN trial with neoadjuvant combined immunotherapy (ipilimumab + nivolumab) achieved an overall response rate of 80% in 10 patients, albeit accompanied by high toxicity.

## Immunotherapy in the metastatic setting

The survival of advanced, unresectable metastatic melanoma has been greatly improved over the last few years. This unprecedented development is related to the introduction of immune checkpoint inhibitors with antibodies against CTLA-4 and PD-1 and targeted therapy with BRAF and MEK inhibitors. The most important studies presented during ECCO 2017 regarded the outcomes of anti-PD-1 immunotherapy in poorly prognostic subgroups of patients.

Reports from the KEYNOTE-006 study with pembrolizumab versus ipilimumab analyzed the outcome of patients with elevated lactate dehydrogenase (LDH) levels at baseline [10]. Progression-free survival (PFS) was better in patients treated with pembrolizumab compared with ipilimumab (normal LDH, median 7.0 months vs. 2.9 months; elevated LDH, median 2.8 months vs. 2.5 months). For overall survival (OS), the median was not reached in either group for normal LDH patients, but in elevated LDH patients, improved survival was observed with pembrolizumab (median 14.7 months vs. 6.2 months). However, the general outcomes for anti-PD-1 monotherapy were unsatisfactory in the group with increased LDH (especially if more than twice the upper limit norms). Similarly, patients with advanced mucosal melanoma treated with pembrolizumab in three studies (KEYNOTE-001, KEYNOTE-002, and KEYNOTE-006 [11]) experienced a median PFS of 2.8 months and median OS of 11.3 months, which were worse than for non-mucosal melanoma patients, yet anti-PD-1 activity was still observed.

## Conclusions

Following the promising results of new systemic therapies in stage IV disease, these are currently being investigated in phase II and III trials in adjuvant and neoadjuvant settings. Neoadjuvant therapy has several potential advantages, including a possibility of decreased surgical morbidity, prevention of micrometastases, personalization of adjuvant therapy based on the response to neoadjuvant therapy, and the opportunity to collect biospecimens for evaluation of the underlying mechanisms of action and drug resistance. In metastatic disease, new strategies are desired for patients with poorer prognostic factors at baseline.
